# High Oestrogen receptor alpha expression correlates with adverse prognosis and promotes metastasis in colorectal cancer

**DOI:** 10.1186/s12964-024-01582-1

**Published:** 2024-03-28

**Authors:** Geriolda Topi, Shakti Ranjan Satapathy, Souvik Ghatak, Karin Hellman, Fredrik Ek, Roger Olsson, Roy Ehrnström, Marie-Louise Lydrup, Anita Sjölander

**Affiliations:** 1 Division of Cell and Experimental Pathology, Department of Translational Medicine, Lund University, Malmö, Sweden; 2 Chemical Biology & Therapeutics Group, Department of Experimental Medical Science, Lund University, Lund, Sweden; 3 Department of Pathology, Skåne University Hospital, Malmö, Sweden; 4 Division of Surgery, Skåne University Hospital, Malmö, Sweden; 5 Department of Endocrinology, Skåne University Hospital, Malmö, Sweden

**Keywords:** Oestrogen receptor alpha, Colorectal cancer, Cysteinyl leukotriene receptors, Metastasis, Zebrafish, PPT, AZD9496

## Abstract

In normal colon tissue, oestrogen receptor alpha (ERα) is expressed at low levels, while oestrogen receptor beta (ERβ) is considered the dominant subtype. However, in colon carcinomas, the ERα/β ratio is often increased, an observation that prompted us to further investigate ERα’s role in colorectal cancer (CRC). Here, we assessed ERα nuclear expression in 351 CRC patients. Among them, 119 exhibited positive ERα nuclear expression, which was significantly higher in cancer tissues than in matched normal tissues. Importantly, patients with positive nuclear ERα expression had a poor prognosis. Furthermore, positive ERα expression correlated with increased levels of the G-protein coupled cysteinyl leukotriene receptor 1 (CysLT_1_R) and nuclear β-catenin, both known tumour promoters. In mouse models, ERα expression was decreased in *Cysltr1*^*−/−*^ CAC (colitis-associated colon cancer) mice but increased in *Apc*^*Min/+*^ mice with wild-type *Cysltr1*. In cell experiments, an ERα-specific agonist (PPT) increased cell survival via WNT/β-catenin signalling. ERα activation also promoted metastasis in a zebrafish xenograft model by affecting the tight junction proteins ZO-1 and Occludin. Pharmacological blockade or siRNA silencing of ERα limited cell survival and metastasis while restoring tight junction protein expression. In conclusion, these findings highlight the potential of ERα as a prognostic marker for CRC and its role in metastasis.

**Supplementary Information:**

The online version contains supplementary material available at 10.1186/s12964-024-01582-1.

## Introduction

Oestrogen receptor alpha (ERα), one of the two primary oestrogen receptors encoded by the *ESR1* gene located on chromosome 6, plays a crucial role in the tumorigenesis of various cancers, such as breast, prostate, uterine, and ovarian cancers [1–4]. In normal colon mucosa, ERα expression is generally low [5, 6]. However, studies have shown an increased ERα/β ratio in colon carcinomas [7, 8]. Despite this discrepancy, there have been limited investigations of the prognostic significance of ERα in colorectal cancer (CRC) patients [9–11]. Therefore, further research is needed to elucidate the prognostic implications of ERα in CRC.

CRC is currently the third most common cancer worldwide, claiming thousands of lives annually [12, 13]. The development of CRC is associated with genetic alterations and inflammation [14, 15]. Mutation of the tumour suppressor gene adenomatous polyposis coli (*APC*) is present in approximately 85% of sporadic CRC cases [16]. Somatic loss of *APC* results in aberrant activation of the Wnt/β-catenin pathway, which is known to play a pivotal role in the development and prognosis of CRC [16, 17]. Additionally, cysteinyl leukotrienes (CysLTs) have been implicated in colon tumorigenesis [18]. Previous studies have investigated the tumour-promoting effects of CysLT_1_R and have demonstrated its association with poor prognosis in CRC patients, as well as its tumour-promoting effects in colon cancer (CC) cell lines and several mouse models [19–21].

In this study, using CRC patient material, animal models, and CC cell lines, we investigated how ERα influences the prognosis of CRC patients and promotes metastasis in CRC. We report that an increase in ERα expression leads to poor outcomes in patients with CRC and that activated ERα expression stimulates the expression of tumour promoters and drives metastasis via regulation of tight junction proteins. Furthermore, we demonstrate that targeting ERα either through pharmacological inhibition or siRNA silencing could decrease the metastatic burden in preclinical models of CC.

## Materials and methods

### Patients

The patients involved in this study were represented from two cohorts: the Kvinno cohort, composed of a total of 333 CRC samples collected between January 1, 2008, and June 30, 2012; and the Malmö cohort, comprising 120 CRC samples collected during 1990. All the samples collected were incorporated into tissue microarrays (TMAs). Details about the study design and patient follow-up for each cohort were provided earlier [22, 23]. Both studies were approved by the Ethics Committee at Lund University. The flowchart of patient inclusion in the study is shown in Supplementary fig. S1.

### Tissue microarray (TMA) and immunohistochemical (IHC) analyses

The tissues from CRC samples incorporated into the TMAs were stained to evaluate the expression of ERα using a mouse anti-ERα antibody cocktail (overnight incubation, pH 6.0) and an anti-ERα monoclonal antibody D-12 (overnight incubation, pH 6.0) as mentioned earlier [24]. Additionally, anti-CysLT_1_R (overnight incubation) and anti-total β-catenin antibodies were used. Immunoreactivity was assessed by two blinded independent investigators (GT and RE) using the immunoreactive score (IRS) calculated as follows: IRS = (staining intensity) × (% of stained cells) [22, 24]. Only the nuclear ERα expression was taken into consideration. All the cores with positive nuclear staining in more than 10% of the cells, regardless of the staining intensity, were considered positive for ERα expression. Tissue cores with less than 10% of positive nuclear staining were considered negative for ERα expression. For antibody dilutions and details, see Table 1.

**Table 1:** List and details of reagents and antibodies used in this study

Reagents or Antibodies	Source	Identifier no.	Dilution	Assay
Anti-ERα antibody (1D5 + 6F11)	Thermo Fisher	MA5–14104	1:50	Immunohistochemistry
Anti-ERα antibody (D-12)	Santa Cruz Biotechnology	sc-8005	1:25	Immunohistochemistry
Anti-CysLT1R antibody	Novus Biologicals	NLS1317	1:100	Immunohistochemistry
Non-phospho (Active) β-Catenin (Ser33/37/Thr41) (D13A1) Rabbit mAb	Cell Signalling	8814	1:100	Immunohistochemistry
Anti-ERα antibody (D-12)	Santa Cruz Biotechnology	sc-8005	1:500	Western blotting
Phospho β-Catenin (Ser33/37/Thr41)	Cell Signalling	9561	1:1000	Western blotting
Non-phospho (Active) β-Catenin (Ser33/37/Thr41) (D13A1) Rabbit mAb	Cell Signalling	8814	1:3000	Western blotting
Total β-Catenin (D10A8) XP® Rabbit mAb	Cell Signalling	8480	1:5000	Western blotting
Anti-ZO-1	Invitrogen	33–9100	1:1000	Western blotting
Anti-GAPDH antibody (0411)	Santa Cruz Biotechnology	sc-47724	1:5000	Western blotting
Anti-ZO-1	Invitrogen	33–9100	1:100	Immunofluorescence
Anti-Occludin (E-5)	Santa Cruz Biotechnology	sc-133256	1:100	Immunofluorescence
Goat anti-mouse IgG (H + L) Cross-Adsorbed Secondary Antibodies Alexa Fluor™488, Alexa Fluor™555	Invitrogen	A-11001, A-21422	1:400	Immunofluorescence
DAPI	Sigma-Aldrich	D9542	5 μg/mL	Immunofluorescence
Vybrant-DiI	Invitrogen	V22885	20 μM	Zebrafish xenograft
PPT	Tocris	1426	40 nM	–
AZD9496	Medchem Tronica	HY-12870	0.3 nM	–

### Cell lines

The human CC cell lines HT-29 and Caco-2 were obtained from the American Type Culture Collection (ATCC, Manassas, Virginia, USA). HT-29 cells were cultured in McCoy’s 5A medium supplemented with 10% foetal bovine serum (FBS), 1% L-glutamine and 100 μg/ml penicillin/streptomycin. Caco-2 cells were maintained in MEM supplemented with 20% foetal bovine serum (FBS), 1% L-glutamine, 1% non-essential amino acids, and 100 μg/ml penicillin/streptomycin. Both cell lines were incubated at 37 °C and 5% CO_2_. Cells were treated for 48 h with the ERα-selective agonist, PPT (40 nM) with or without treatment with the ERα-selective antagonist AZD9496 (0.3 nM) 30 min before PPT stimulation.

### Quantitative real-time PCR

The isolation of total RNA from CC cells was performed using RNeasy Mini Kit (Qiagen, Hilden, Germany), and first-strand cDNA synthesis was accomplished using a cDNA synthesis kit (Invitrogen, Carlsbad, California, USA) [25–28]. The TaqMan probes used were specific for *ESR1* (ERα, Hs01046816_m1), *CYSLTR1* (CysLT_1_R, Hs00272624-s1), and *CTNNB1* (β-catenin, Hs00991818_ml). The samples were analysed, expression levels were normalized to those of the endogenous housekeeping gene *HPRT1* (Hs99999909_m1), and fold changes were quantified with the 2-ΔΔCt method using MxPro software (Agilent Technologies, Santa Clara, CA, USA).

### Western blotting

Western blotting was performed based on a protocol described previously with minor modifications [25–29]. The following primary antibodies were used: anti-ERα, anti-phospho-β-catenin (Ser45/Thr41), anti-non-phospho (active)-β-catenin, and anti-total β-catenin. Anti-α-tubulin and anti-glyceraldehyde 3-phosphate dehydrogenase (GAPDH) antibodies were used as loading control antibodies as indicated in the figures. Visualization of protein expression was performed with enhanced chemiluminescence (ECL) reagents Millipore with a Bio-Rad ChemiDoc™ imaging system (Hercules, CA, USA), and densitometric analysis was performed using Bio-Rad Image Lab software. For antibody dilutions and details (see Table 1).

#### *siRNA* transfection in colon cancer cells

An *ESR1*-specific siRNA (*siESR1*) was employed to study ERα specificity in functional assays using CC cells in accordance with a previously reported protocol [27].

### Colonosphere model

HT-29 and Caco-2 CC cell-derived colonospheres were generated as described previously [25–27].

### Immunofluorescence analysis

Immunofluorescence analysis was performed in HT-29 and Caco-2 cells to visualize the expression of the tight junction proteins ZO-1 and Occludin as previously described [29]. Fluorescence images were captured with a Zeiss LSM 700 confocal microscope (Carl Zeiss Microscopy GmbH, Jena, Germany) using a 63× oil objective and analysed using LSM ZEN Blue software.

Immunofluorescence staining in colonospheres was performed by adopting a protocol designed for organoids with some modifications [30]. Briefly, CC cells were used to generate colonospheres as described previously [25–27]. After 72 h, colonospheres were harvested and cultured in Matrigel in eight-well glass-bottom chamber slides (Ibidi, GmbH, Germany). After 24 h, the medium was removed from each chamber, and the colonosphere embedded in the Matrigel was washed with 1x PBS and fixed with 4% PFA for 1 h at room temperature (RT). After fixation, the colonospheres were washed with 1x PBS and permeabilized with 0.3% Triton X-100 for 15 min at RT. Blocking was performed with 5% BSA prepared in PBS containing 0.1% Tween 20 overnight at 4 °C. Colonospheres were incubated with the primary antibody (see Table 1 for dilutions) overnight at 4 °C prior to washing with PBS containing 0.1% Tween 20. Next, incubation with a secondary antibody combined with a nuclear stain was performed for 4 h at RT prior to washing with 1x PBS containing 0.1% Tween 20. Colonospheres were mounted using Dako mounting medium (Dako, Denmark), and images were acquired with a confocal microscope (Zeiss, Germany).

### Colon cancer mouse models

We evaluated the expression of ERα in two different CC mouse models. C57BL/6 J-*Apc*^*Min/+*^ mice (The Jackson Laboratory, Bar Harbor, Maine, USA) and in a CAC model with *Cysltr1* gene disruption on the C57BL/6 N background [31]. The experimental design for the CAC model is described in detail earlier [32]. Tissues from these mice were stained with an anti-ERα antibody (1D5 + 6F11, 1:50) to evaluate ERα expression. Five mice per genotype were included, and four different sections per mouse were evaluated.

### Zebrafish xenograft model

The role of ERα expression in CC cell metastasis was validated using the zebrafish xenograft model [25–27, 33]. HT-29 CC cells were treated with PPT with or without AZD9496 for 48 h, labelled with Vybrant DiI and microinjected into the perivitelline space of the 2 days post-fertilization zebrafish embryos. *siCTRL-* or *siESR1*-transfected cells were treated with PPT for 48 h before DiI labelling and were injected into the embryos. After 48 h, the metastatic spread of CC cells into the tail veins of the embryos was monitored and photographed using a Nikon fluorescence microscope (Nikon Eclipse 80i, USA). The mean fluorescence intensity (MFI) of the metastatic cells was measured using ImageJ software (NIH, USA). The zebrafish housing and maintenance were as described before [34].

### Statistical analysis

Continuous variables were compared using the Mann-Whitney U test or Student’s *t* test as indicated, and categorical variables were compared using the chi-square test. Kaplan–Meier- survival curves were compared with the log-rank test. Statistical analyses were performed using SPSS version 23.0 (SPSS, IBM, Armonk, NY, USA) and GraphPad Prism version 9 (GraphPad Software, Inc., San Diego, CA, USA). All tests were two sided, and *P* values < 0.05 were considered to indicate a statistically significant difference.

### Public CRC dataset analysis

We also used public CRC patient data. The mRNA expression based microarray data from the GSE39582 dataset were downloaded. ERα gene expression in this public database was categorised into high ERα and low ERα. Data for a total of 566 patients were included in the study. The mRNA expression data in the dataset were normalized using the TMM method and were log2 transformed for further analysis.

## Results

### ERα expression and its prognostic association in colorectal cancer patients

**Fig. 1:**
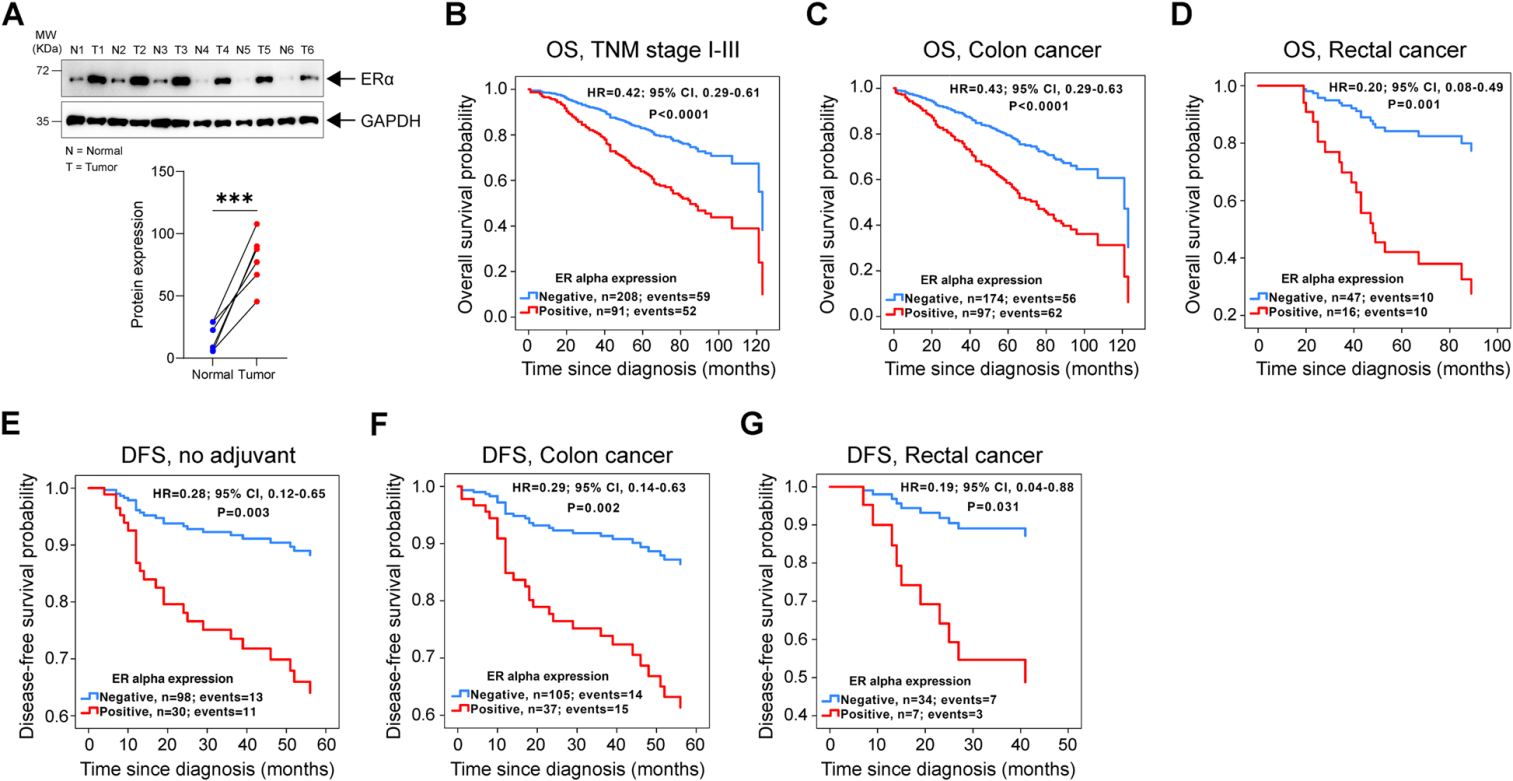
ERα expression and its prognostic association in colorectal cancer (CRC) patients. **A** Western blot of ERα protein expression in six paired colon cancer (CC) patient samples of normal (N) and tumour (T) colon tissue. GAPDH was used as a loading control. Graph showing the densitometric analysis of ERα protein expression compared between normal (N) and tumour (T) tissues. Kaplan-Meier survival curves for OS: **B** multivariate model for CRC patients with stage I-III disease, *n* = 299; **C** multivariate model for patients with colon cancer, *n* = 271; and **D** multivariate model for patients with rectal cancer, *n* = 63. Kaplan-Meier survival curves for DFS: **E** multivariate model for patients who did not receive adjuvant treatment after surgery, *n* = 128; **F** multivariate model for patients with colon cancer, *n* = 142; and **G** multivariate model for patients with rectal cancer, *n* = 41. *P* values < 0.5 were considered significant and determined by the log-rank test

Recently, we reported that *KRAS* oncogenic mutation correlates with positive ERα expression in CC patients [24]. However, the involvement of ERα in CC growth and metastasis is not yet clear. Here, using paired normal and tumour samples of CRC patients, we found consistently elevated expression of ERα in the tumour tissue compared to the normal mucosal tissue (Fig. 1A). Previously, we reported that high ERα expression in CRC patients is associated with poor overall survival (OS) [24]. Here, we combined the same cohort (Kvinno cohort, *n* = 270) with another cohort of patients with CRC (Malmö cohort, *n* = 67) and grouped the patients based on ERα expression (positive or negative) (Supplementary Fig. S1). After excluding patients from both cohorts with metastatic disease or an unknown recurrence status or date, 233 patients were available for disease free survival (DFS) analysis. The median follow-up times for the Kvinno cohort were 5.8 years for OS and 5 years for DFS. For the Malmö cohort, the median follow-up time for OS was 10 years. To investigate the prognostic role of ERα expression in CRC patients, we performed Cox regression analysis and evaluated OS and DFS. Patients with negative ERα expression had a significantly reduced risk for overall mortality of 61% (HR, 0.39; CI, 0.28–0.55) after adjustment for age and TNM stage (Fig. 1B). This result was consistent in the subgroups of patients with stage I-III CRC disease and colon cancer and, interestingly, also in the subgroup of patients with rectal cancer, in which negative ERα expression showed the greatest benefit, with a reduction in overall mortality of 80% (Fig. 1B-D). Similar results were obtained for patients who did not receive any adjuvant treatment after CRC surgery, for both patients with colon or rectal cancer (Fig. 1E-G). Together these data indicates that high ERα expression is associated with poor prognosis in CRC patients.

### ERα expression positively correlates with tumour promoter expression in colon cancer

**Fig. 2:**
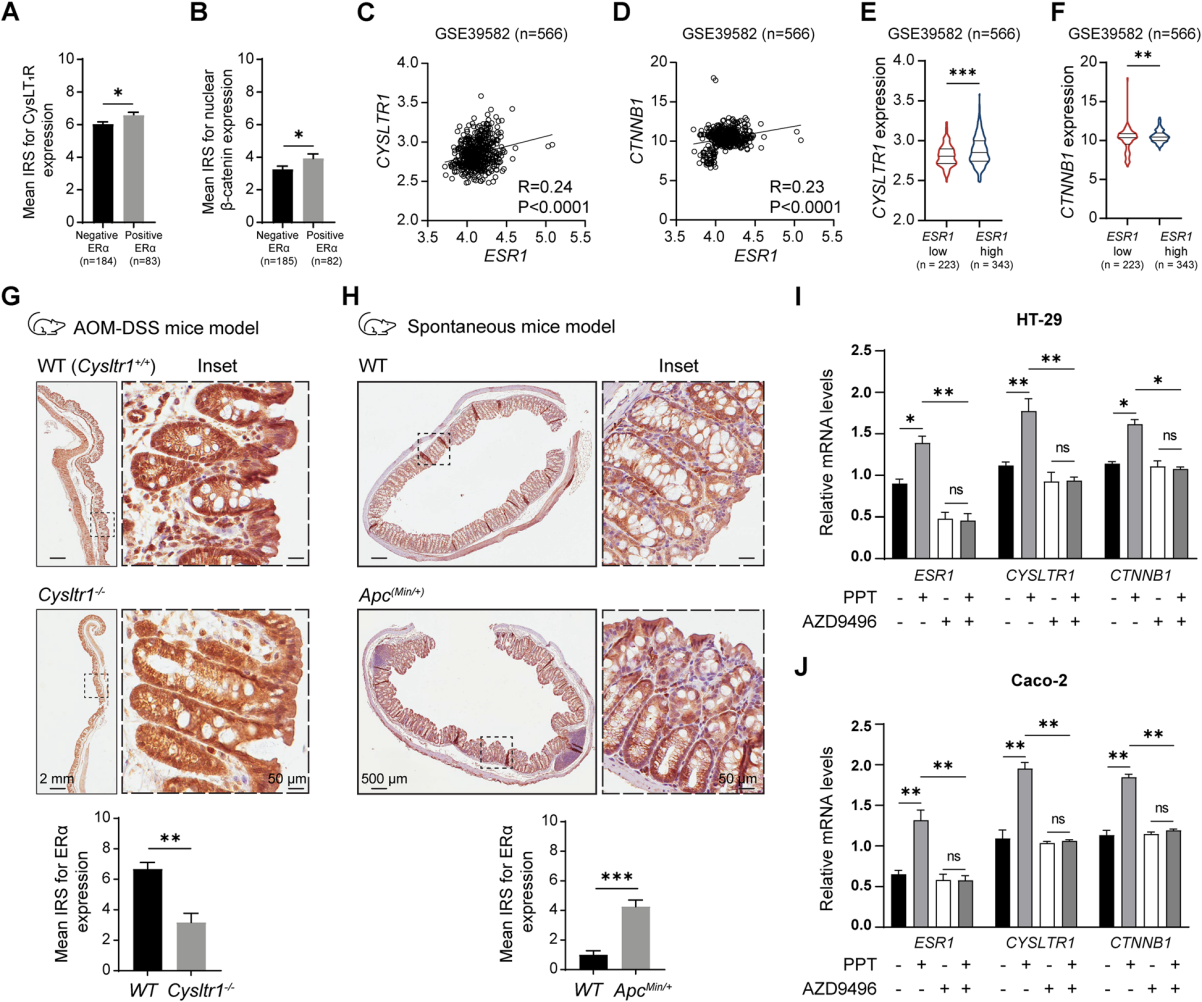
ERα expression positively correlates with tumour promoter expression in colon cancer. Mean immuno-reactive score (IRS) for (**A**) Cysteinyl leukotriene receptor 1 (CysLT_1_R) and (**B**) nuclear β-catenin expression in colorectal cancer (CRC) patients (*n* = 267) with negative and positive ERα expression. XY scatter plots of the mRNA levels of (**C**) ERα (*ESR1*) and CysLT_1_R (*CYSLTR1*) and (**D**) ERα (*ESR1*) and β-catenin (*CTNNB1*) in the GSE39582 public dataset (*n* = 566) of CRC patients. Violin plots showing the mRNA expression of (**E**) *CYSLTR1* and (**F**) *CTNNB1* in CRC patients with low or high *ESR1* expression. **G** Immunohistochemical (IHC) images of ERα expression in the colons of wild-type (*WT*) and *Cysltr1* knockout (*Cysltr1*^−/−^) mice in a colitis-associated colon cancer (CAC) mouse model (n = 5). Bar graph showing the IRS of ERα expression compared between the *WT* and *Cysltr1*^*−/−*^ mouse groups. **H** IHC images of ERα expression in the colons of *WT* and *Apc*^*Min/+*^ mice, *n* = 5. Bar graph showing the IRS of ERα expression in the *WT* and *Cysltr1*^*−/−*^ mouse groups. For both mouse models, four random regions of interest (ROIs; marked with dotted lines) in colon tissue were evaluated for each mouse. Representative images of one ROI are shown as insets. The scale bars represent 2 mm (**G**) and 500 μm (**H**) in the image of the whole colon and 50 μm in the zoomed insets. *P* values were calculated using an unpaired Student’s *t* test for the bar graphs in G and H. Relative mRNA expression levels of *ESR1*, *CYSLTR1*, and *CTNNB1* in (**I**) HT-29 and (**J**) Caco-2 CC cells after treatment with PPT (ERα specific agonist, 40 nM) or AZD9496 (ERα specific antagonist, 0.3 nM for 30 min) alone or in combination of PPT (40 nM) with AZD9496 (0.3 nM for 30 min before the PPT treatment). The data are presented as the mean ± SEM (*n* = 3 independent experiments). P values < 0.5 were considered significant were calculated using an unpaired Student’s *t* test

In this cohort, we previously showed that the poor prognosis of the included patients was associated with high CysLT_1_R and nuclear β-catenin expression. These two tumour promoters in colon cancer are known to act through a CysLT_1_R/Wnt-β-catenin signalling axis [25, 28, 32, 35]. Here, we tested for possible associations between ERα and CysLT_1_R and/or β-catenin. As expected, we noted higher expression of CysLT_1_R and nuclear β-catenin in patients with positive ERα expression (*n* = 185) compared to patients with negative ERα expression (*n* = 82) (Fig. 2A, B). This finding was further validated by analysis of a public CRC dataset (GSE39582, *n* = 566), which revealed a significant positive correlation between the mRNA level of *CYSLTR1* or *CTNNB1* (β-catenin) and *ESR1* (ERα) (Fig. 2C, D). In addition, patients with high *ESR1* expression showed higher transcript levels of both *CYSLTR1* and *CTNNB1* (Fig. 2E, F).

Next, we used both the colitis-associated colon cancer (CAC) mouse model and spontaneous CC mouse model (*Apc*^*Min/+*^) with either the functional presence or functional absence of *Cysltr1* (n = five mice per genotype) (Fig. 2G, H). *Cysltr1*^*−/−*^ mice are reported to exhibit a less aggressive tumour phenotype, with fewer polyps/tumours in their colon [32, 35]. Colon tissue sections from mice lacking functional *Cysltr1* (*Cysltr1*^*−/−*^) showed two-fold reduced ERα expression compared to those from *WT* mice (Fig. 2G). On the other hand, we found that *Apc*^*Min/+*^ mice, which have higher amount of β-catenin accumulation in the nucleus due to the *Apc* mutation and are reported to develop more and larger intestinal polyps [36], had >two-fold higher nuclear ERα expression than mice with wild-type *Apc* (Fig. 2H).

Furthermore, we evaluated the basal mRNA levels of ERα expression in different CC cell lines, using the MCF7 breast cancer cell line, with very high expression of ERα, as a positive control (data not shown). Based on these results we used the ERα expressing cell lines HT-29 and Caco-2 cells for further studies. We pharmacologically induced or blocked ERα expression in HT-29 and Caco-2 cells by treatment with the ERα-selective agonist PPT alone or ERα-specific antagonist AZD9496 or combination of both (AZD9496 + PPT) (see Materials and Methods for experimental details). We observed a more than 30–50% increase in the mRNA expression levels of both *CYSLTR1* and *CTNNB1* with PPT treatment, and this increase was restricted after AZD9496 alone treatment or in PPT stimulated cells pre-treated with AZD9496 (PPT + AZD9496) in both HT-29 and Caco-2 cells (Fig. 2I, J). No statistical significance was noted between AZD9496 or AZD9496 + PPT experimental groups (Fig. 2I, J). Taken together, these results indicate a direct involvement of ERα in promoting the expression of the tumorigenic markers CysLT_1_R and β-catenin in CC.

### ERα activation in colon cancer cells promotes survival

**Fig. 3:**
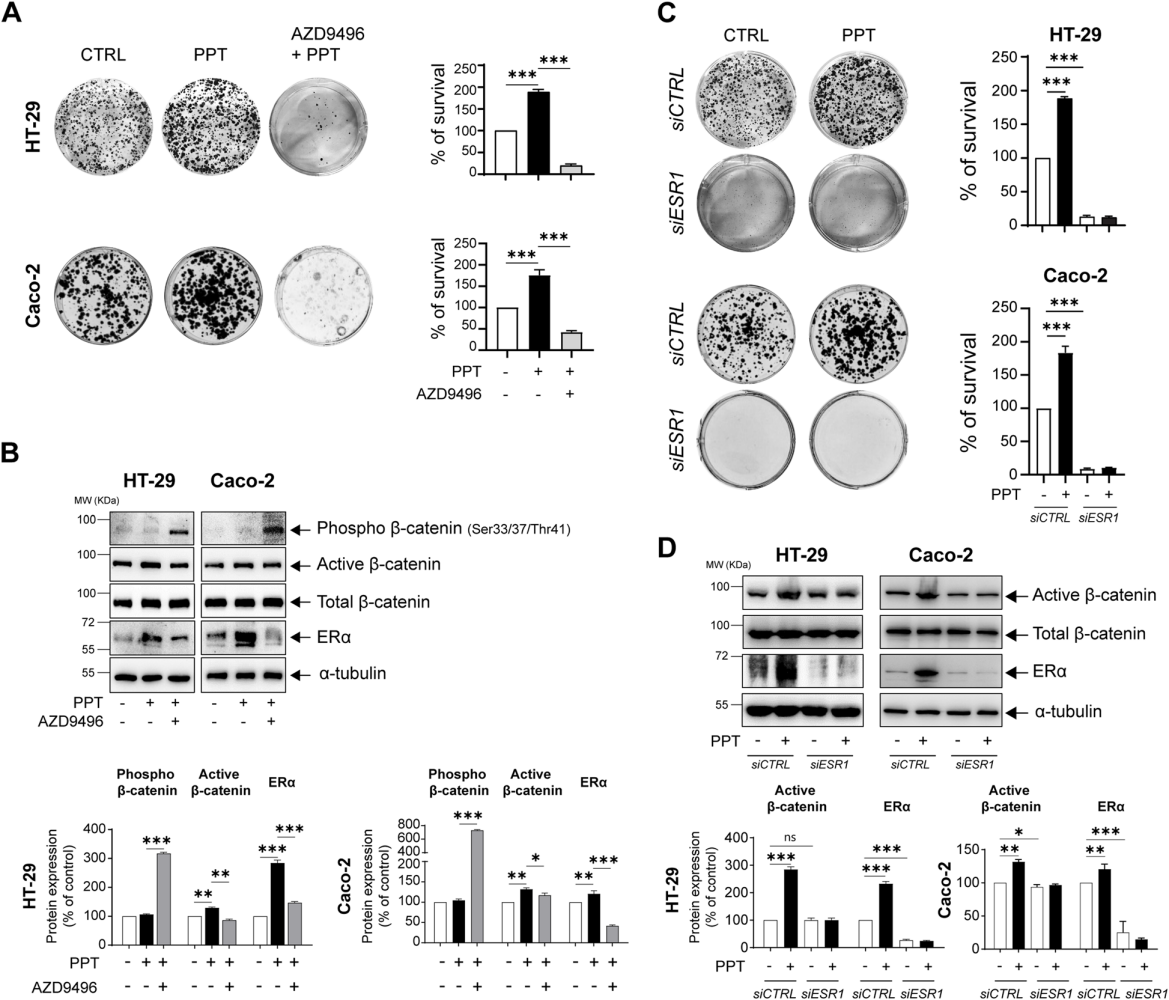
ERα activation in colon cancer cells promotes survival. **A** Alterations in the colonies formed by HT-29 and Caco-2 colon cancer (CC) cells treated with PPT (40 nM) alone for 48 h or in combination with AZD9496 (0.3 nM, for 30 min before PPT treatment). Bar graphs show the percentage of survival and are representative of *n* = 3 independent experiments. **B** Western blots showing the protein levels of phospho-β-catenin (Ser33/37/Thr41), non-phospho (active)-β-catenin, total β-catenin, and ERα in HT-29 and Caco-2 cells untreated or treated with PPT (40 nM) alone or in combination with AZD9496 (0.3 nM, for 30 min). Graphs showing the densitometric analysis of alterations in phospho- and non-phospho (active)-β-catenin and ERα protein levels as percentages of the loading control (α-tubulin). The blots are representative of n = 3 independent experiments. **C** Alterations in the colonies formed by HT-29 and Caco-2 cells transfected with either *siCTRL* or *siESR1* prior to PPT (40 nM) treatment for 48 h. The graphs show the percentage of survival in each group. **D** Western blots showing the protein levels of non-phospho (active)-β-catenin, total β-catenin, and ERα in both HT-29 and Caco-2 cells transfected with either *siCTRL* or *siESR1* prior to PPT (40 nM) treatment. Graphs showing the densitometric analysis of alterations in ERα and non-phospho (active)-β-catenin protein levels as percentages of the loading control (α-tubulin). The blots are representative of n = 3 independent experiments. The data are presented as the means ± SEMs. *P* values < 0.5 were considered significant calculated using an unpaired Student’s *t* test

WNT/β-catenin signalling in CC is known to support tumour growth via nuclear β-catenin. Based on the observations in Fig. 2B and H, we next sought to investigate whether activation of ERα can promote cell survival via the WNT/β-catenin pathway. Indeed, we observed a 2-fold increase in the number of colonies formed by both HT-29 and Caco-2 CC cells treated with PPT compared to untreated control cells, and the number of colonies formed by cells pretreated with AZD9496 was substantially reduced (Fig. 3A). Furthermore, western blot analysis of HT-29 and Caco-2 cells stimulated with PPT showed increased levels of non-phosphorylated active β-catenin compared to unstimulated cells (Fig. 3B). Interestingly, the increase in the β-catenin level was inhibited in cells pretreated with AZD9496 prior to PPT stimulation, possibly due to the significant increase in phosphorylated β-catenin (three-fold for HT-29 and six-fold for Caco-2 cells), which is known to be rapidly degraded by ubiquitination.

To further validate the involvement of ERα in CC cell survival, we employed *ESR1* siRNA to silence functional ERα, and the results were compared with those in *siCTRL*-transfected cells (Fig. 3C, D). Interestingly, we noted a significantly reduced colony number in the *siESR1*-transfected cell group compared to the *siCTRL*-transfected cell group (Fig. 3C). Treatment with PPT did not significantly affect the colony number in the *siESR1*-transfected cell group. Moreover, the whole-cell lysates of HT-29 and Caco-2 cells transfected with *siESR1* showed a reduction in the level of active β-catenin, supporting the earlier observation of the increase in active β-catenin in PPT-treated CC cells (Fig. 3D).

### ERα promotes colon cancer cell metastasis by downregulating tight junction proteins

**Fig. 4:**
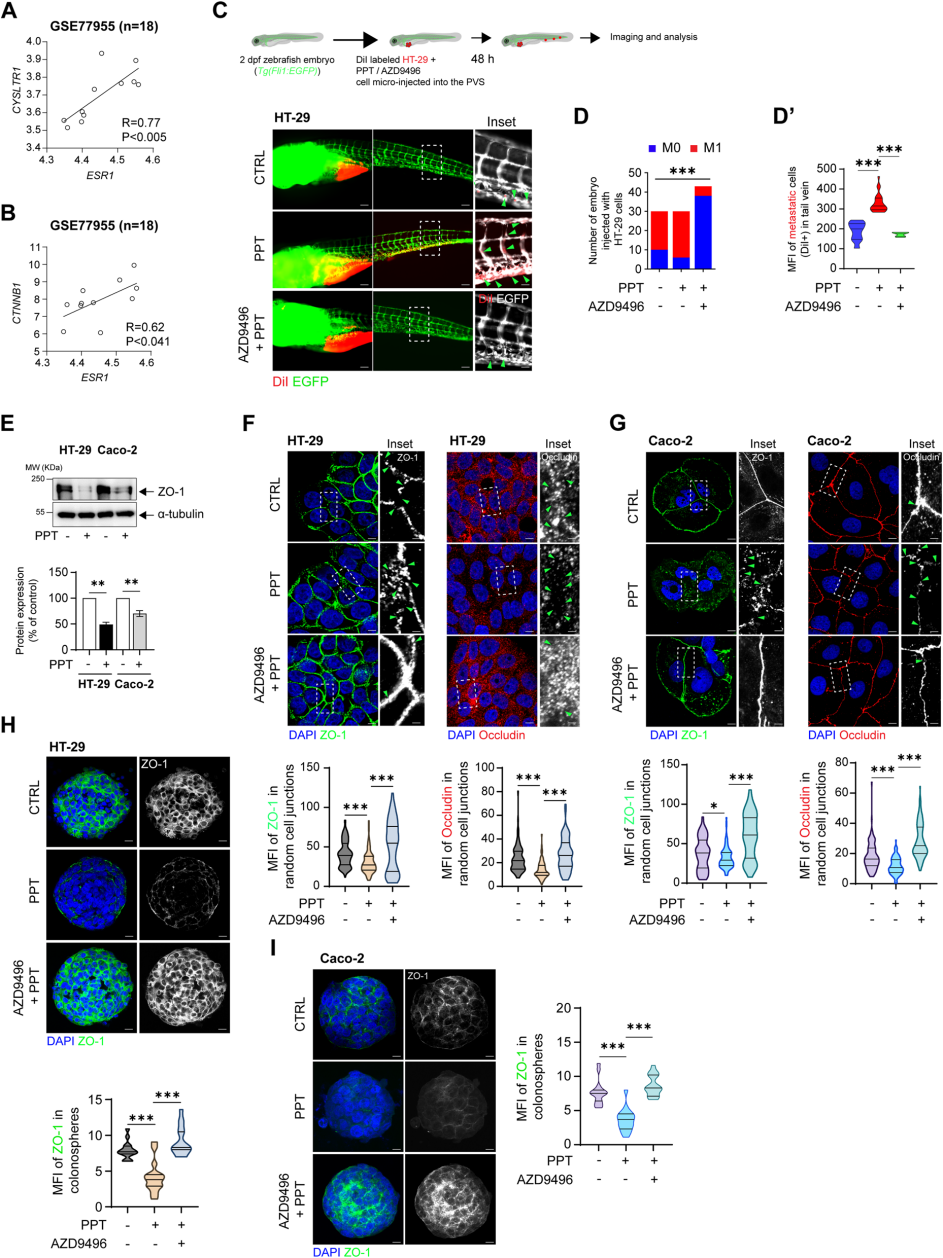
Activation of ERα promotes colon cancer cell metastasis. An external dataset composed of data for CC patients with liver metastasis (GSE77955, *n* = 18) was used to analyse the correlations of CysLT_1_R (*CYSLTR1*) and β-catenin (*CTNNB1*) with ERα (*ESR1*). The scatter plots show the positive correlations between *ESR1* and both (**A**) *CYSLTR1* and (**B**) *CTNNB1*. **C** Schematic cartoon showing the zebrafish embryo-based colon cancer metastasis model. DiI-labelled HT-29 cells left untreated or treated with PPT alone or in combination with AZD9496 were injected into the perivitelline space of 2 dpf zebrafish embryos, and the embryos were incubated for 48 h. Images showing the metastatic spread of HT-29 cells in the tail veins of zebrafish embryos in each group (CTRL, *n* = 30; PPT, n = 30; AZD9496 + PPT, *n* = 43). Scale bars: full-size images; 10 μm, insets; 2 μm. The insets show the regions enclosed in the dotted lines in the full-size tail images. The arrows point to the metastatic foci and transendothelial migration of cancer cells. **D** Graphs showing the number of embryos with (M1, mets) or without (M0, no mets) metastasis in each group and **D′,** quantification of tail vein metastasis using the mean fluorescence intensity (MFI) of the embryos with metastasis. **E** Western blots showing the expression of the tight junction protein ZO-1 in HT-29 and Caco-2 cells treated with PPT (40 nM) alone or in combination with AZD9496 (0.3 nM, 30 min). Graph showing the densitometric analysis of alterations in protein expression as a percentage of the loading control (α-tubulin). The blots are representative of n = 3 independent experiments. For the bar graphs, unpaired t-test was used. **F** Immunofluorescence analysis of ZO-1 and Occludin expression in HT-29 cells treated with PPT (40 nM) alone or in combination with AZD9496 (0.3 nM, 30 min). Greyscale images (insets) showing a representative region of interest (dotted line) for ZO-1 and Occludin staining. Scale bars: full-size images; 5 μm, insets; 1 μm. Violin plots showing the mean fluorescence intensity of ZO-1 (CTRL, *n* = 116; PPT, *n* = 105, AZD9496 + PPT, *n* = 107) and Occludin in random cell-cell junctions (CTRL, *n* = 103; PPT, *n* = 110, AZD9496 + PPT, *n* = 108). *P* values were calculated with unpaired Student’s *t* test. The arrows indicate gaps in ZO-1 expression. **G** Immunofluorescence analysis of ZO-1 and Occludin expression in Caco-2 cells treated with PPT (40 nM) alone or in combination with AZD9496 (0.3 nM, 30 min). Greyscale images (insets) showing representative regions of interest for ZO-1 and Occludin staining. Scale bars: full-size images; 5 μm, insets; 1 μm. Violin plots showing the mean fluorescence intensity of ZO-1 (CTRL, *n* = 108; PPT, *n* = 116, AZD9496 + PPT, *n* = 105) and Occludin (CTRL, n = 105; PPT, *n* = 115, AZD9496 + PPT, n = 116) in random cell-cell junctions. The arrows indicate gaps in ZO-1 or Occludin expression. **H** Immunofluorescence analysis of ZO-1 in HT-29 cell-derived colonospheres treated with PPT (40 nM) alone or in combination with AZD9496 (0.3 nM, 30 min). Scale bars: 10 μm. Violin plot showing the mean fluorescence intensity of ZO-1 in random colonospheres (CTRL, *n* = 31; PPT, *n* = 28, AZD9496 + PPT, *n* = 30). The arrows indicate ZO-1 expression in the disseminated cells from the colonospheres. **I** Immunofluorescence analysis of ZO-1 in Caco-2 CC cell-derived colonospheres treated with PPT (40 nM) alone or in combination with AZD9496 (0.3 nM, 30 min). Scale bars: 10 μm. Violin plot showing the mean fluorescence intensity of ZO-1 in random colonospheres (CTRL, *n* = 29; PPT, *n* = 26, AZD9496 + PPT, n = 28). The data are presented as the mean ± SEM of three experiments. *P* values < 0.5 were considered significant were calculated using the chi-square test in D and an unpaired Student’s *t* test in D′-I

As reported earlier, ERβ activation could reduce metastasis in CC, with a reduced number of migrated cells observed in vitro and in vivo [27]*.* Here, we intended to explore the metastatic potential of ERα, which is known to act in an opposite manner in breast cancer [37]. We used a public dataset of CC patients with liver metastasis (*n* = 18) and found a strong and significant positive correlation between *CYSLTR1* and *ESR1* (R = 0.77, *p* < 0.005) as well as between *CTNNB1* and *ESR1* expression (R = 0.62, *p* < 0.041) (Fig. 4A, B). Next, we tested the impact of ERα activation on the migration capacity of CC cells in vitro using wound-healing assay (Supplementary Fig. S2A). HT-29 cells stimulated with PPT showed more than 2-fold increase in the wound closure compared to unstimulated control. Interestingly, cells pre-treated with AZD9496 showed reduced percentage (5-fold) of wound closure compared to the PPT treated cells suggesting an involvement of ERα in the migration of CC cells. Next, to validate this observation in vivo, we used a transgenic zebrafish xenograft model established with HT-29 cells, interestingly, we observed a higher number of embryos with metastasis as well as an increased metastatic burden in the tail veins of the embryos in the group injected with PPT-treated HT-29 cells (M1 = 24; M0 = 6) compared to the group injected with untreated HT-29 cells (M1 = 10; M0 = 20) (Fig. 4C, D). The increase in metastasis was inhibited in the embryos injected with AZD9496 + PPT-treated HT-29 cells (M1 = 5; M0 = 38). PPT treatment not only resulted in a greater number of embryos with metastasis but also was able to establish a high metastatic burden, as evidenced by quantification of the mean fluorescence intensity (MFI) (mean MFI in the CTRL group = 187; PPT group = 330; AZD9496 + PPT group = 172) (Fig. 4D’).

Cancer cell dissemination due to loss of cell-cell junctions is a key and early step in metastasis. Tight junction proteins are responsible for maintaining physical connections between epithelial cells. Important among tight junction proteins is the ZO-1/Occludin complex [38]. Therefore, we next sought to determine whether ERα-mediated metastasis in CC occurs via disruption of tight junctions with a possible change of the ZO-1 complex. Indeed, we found a decrease in ZO-1 expression in HT-29 (50%) and Caco-2 (40%) cells treated with PPT (Fig. 4E). This was further supported by immunofluorescence staining of HT-29 and Caco-2 cells, where it was clearly evident that there was disruption of ZO-1 expression in cells after PPT treatment, while in both untreated and AZD9496-treated cells, ZO-1 expression remained unchanged (Fig. 4F, G). Furthermore, Occludin, a member of the ZO-1 complex, was expressed at significantly lower levels in PPT-treated HT-29 or Caco-2 cells owing to disrupted cell-cell junctions (Fig. 4F, G). Moreover, analysis of ZO-1 expression in HT-29 and Caco-2 cell-derived colonospheres validated the above observations (Fig. 4H, I). These results indicate the metastasis-promoting role of ERα induction in CC cells and the beneficial effects of antagonizing ERα expression by treatment with a selective antagonist such as AZD9496.

### The functional absence of ERα inhibits colon cancer cell metastasis

Next, we tested the effect of the functional absence of *ESR1* on colon cancer metastasis in vitro as well as in vivo. Wound healing assay performed in the *siCTRL* or *siESR1* transfected cells with or without PPT treatment showed > 2-fold increase in wound closure in the *siCTRL* group treated with PPT (Supplementary fig. S2B), while *siESR1* transfected cells failed to show significant wound closure even after PPT treatment.

**Fig. 5:**
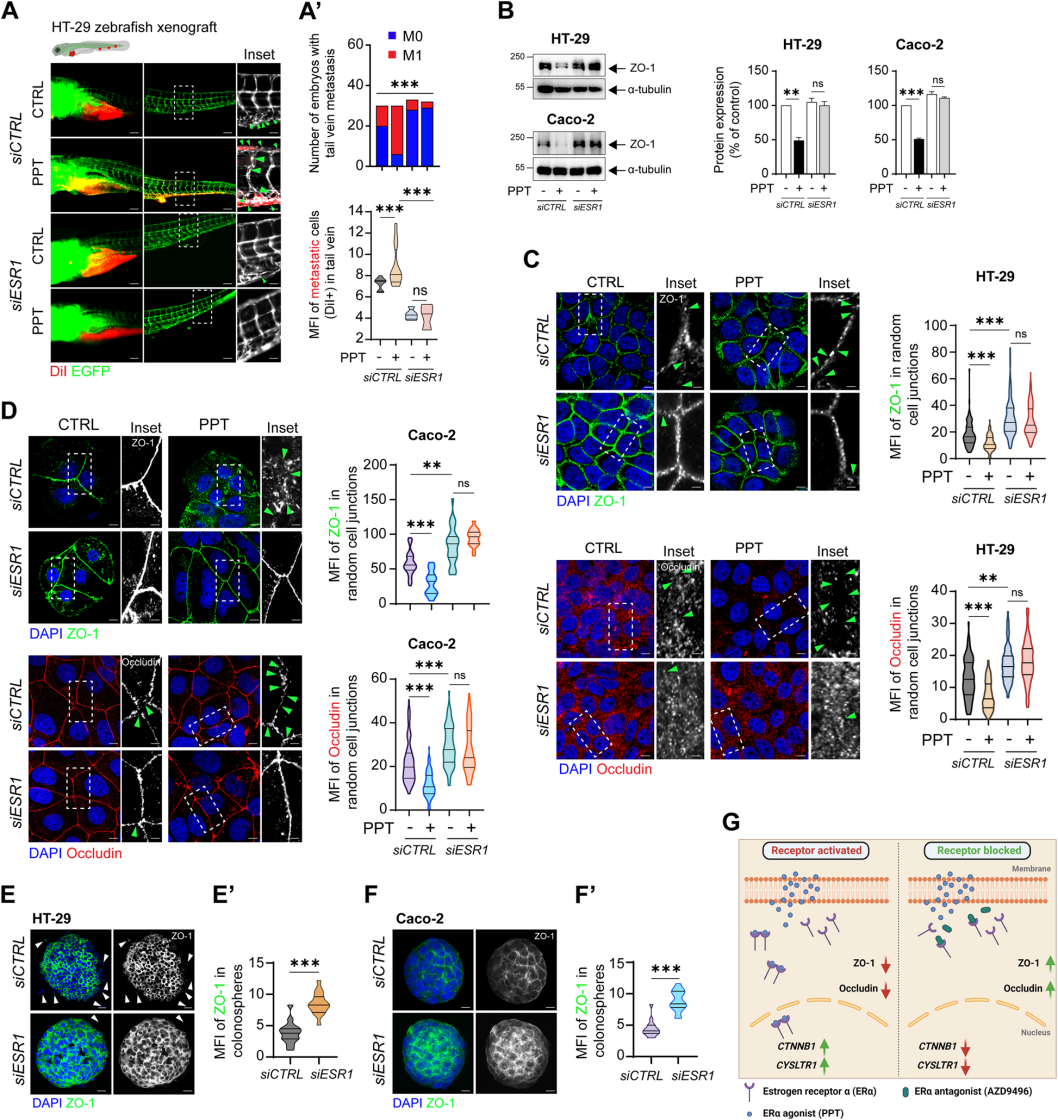
Functional absence of ERα inhibits colon cancer cell metastasis. DiI-labelled HT-29 cells transfected with either *siCTRL* or *siESR1* and treated with or without PPT for 48 h were injected into the perivitelline space of 2 dpf zebrafish embryos, and the embryos were incubated for 48 h. **A** Images showing the metastatic spread of HT-29 cells in the tail veins of zebrafish embryos in each group (*siCTRL*; CTRL, *n* = 30; PPT, n = 30; *siESR1*; CTRL, *n* = 33, PPT, *n* = 32). Graphs showing **A**’, the number of embryos with (M1, mets) or without metastasis (M0, no mets) in each group and **A**”, the quantification of tail vein metastasis using the mean fluorescence intensity (MFI) of the embryos with metastasis (M1 group). Scale bars: full-size images; 10 μm, insets; 2 μm. The insets show the regions enclosed in the dotted lines in the full-size tail images. The arrows point to the metastatic foci and transendothelial migration of cancer cells. **B** Western blots showing the expression of the tight junction protein ZO-1 in HT-29 and Caco-2 cells transfected with either *siCTRL* or *siESR1* prior to PPT (40 nM) treatment. Graphs showing the densitometric analysis of alterations in protein expression as a percentage of the loading control (α-tubulin). The blots are representative of n = 3 independent experiments. **C** Immunofluorescence analysis of ZO-1 and Occludin expression in HT-29 cells transfected with either *siCTRL* or *siESR1* prior to treatment with the ERα agonist PPT (40 nM). Greyscale images (insets) showing representative regions of interest for ZO-1 and Occludin staining. Scale bars: full-size images; 5 μm, insets; 1 μm. Violin plots showing the mean fluorescence intensity of ZO-1 (*siCTRL* (CTRL, *n* = 105; PPT, n = 115), *siESR1* (CTRL, *n* = 108; PPT, n = 116)) and Occludin (*siCTRL* (CTRL, n = 105; PPT, n = 105), *siESR1* (CTRL, *n* = 107; PPT, *n* = 102)) in random cell-cell junctions. The arrows indicate gaps in ZO-1 or Occludin expression. **D** Immunofluorescence analysis of ZO-1 and Occludin expression in Caco-2 cells transfected with either *siCTRL* or *siESR1* prior to treatment with the ERα agonist PPT (40 nM). Greyscale images (insets) showing representative regions of interest for ZO-1 and Occludin staining. Scale bars: full-size images; 5 μm, insets; 1 μm. Violin plots showing the mean fluorescence intensity of ZO-1 and Occludin in random cell junctions. For ZO-1 staining in the *siCTRL*-transfected group (CTRL, n = 105; PPT, n = 105) and in the *siESR1*-transfected group (CTRL, n = 108; PPT, n = 105), random cell junctions were evaluated. For Occludin staining in the *siCTRL*-transfected group (CTRL, n = 105; PPT, n = 105) and in the *siESR1*-transfected group (CTRL, n = 102; PPT, n = 108), random cell junctions were evaluated. The arrows indicate gaps in ZO-1 or Occludin expression. Immunofluorescence analysis of ZO-1 in colonospheres derived from either *siCTRL* or *siESR1* transfected (**E**) HT-29 and (**F**) Caco-2 cells. Scale bars: 10 μm. Violin plots showing the mean fluorescence intensity of ZO-1 in random (**E’**) HT-29 (*siCTRL*, n = 30; *siESR1*, n = 32) or (**F′**) Caco-2 (*siCTRL*, *n* = 28; *siESR1*, *n* = 31) colonospheres. The MFIs of the indicated proteins were measured using ImageJ software (NIH, USA). **G** Graphical representation of the summary of the study. Upon binding to the agonist PPT, ERα dimerizes and shuttles into the nucleus. This upregulates the transcription of *CYSLTR1* and *CTNNB1*. In addition, it promotes metastasis by disrupting the tight junction proteins ZO-1 and Occludin. However, blocking the binding of PPT to ERα by employing an antagonist, AZD9496, prevents the activation and hence the dimerization of the receptor. This further leads to downregulation of *CYSLTR1* and *CTNNB1* and upregulation of the tight junction proteins ZO-1 and Occludin. The data are presented as the mean ± SEM of three experiments. *P* values were calculated with the chi-square test for A’ and unpaired Student’s *t* test for A”, B-F

While injection of PPT-treated *siCTRL*-transfected cells resulted in metastasis in a higher number of embryos (embryos with metastasis (M1) = 3; embryos with no metastasis (M0) = 29), *siESR1* transfection failed to result in metastasis even after PPT treatment (M1 = 5, M0 = 24) (Fig. 5A, A’). Zebrafish embryos injected with HT-29 cells transfected with *siESR1* showed reduced tail vein metastasis compared to embryos injected with *siCTRL*-transfected HT-29 cells, with a 3-fold reduction in the MFI indicating the metastatic burden in embryos with metastasis (Fig. 5A’).

We observed a reduced number of embryos with tail metastasis upon *ESR1* silencing (*siESR1*) (M1, *n* = 5) (Fig. 5A), we posited that cells, with *ESR1* gene silencing, might be tightly adhered to each other, indicating increased ZO-1 expression. Initially, we employed western blotting to assess ZO-1 expression under the mentioned experimental conditions. As observed in Fig. 4; in Fig. 5B, we noted reduced ZO-1 expression in whole-cell lysates of *siCTRL*-transfected HT-29 (50%) and Caco-2 (50%) cells after PPT treatment. Surprisingly, we found a similar expression in the *siESR1*-transfected control group compared to the *siCTRL*-transfected control group in HT-29 cells but not in Caco-2 cells (Fig. 5B). However, ZO-1 expression in *siESR1*-transfected HT-29 and Caco-2 CC cells remained unaffected even after PPT treatment (Fig. 5B). To validate this, we performed immunofluorescence analysis in HT-29 and Caco-2 CC cells for visual confirmation. Indeed, in the immunofluorescence images, elevated ZO-1 expression was evident at the ‘cell-cell junctions’ of *siESR1* transfected HT-29 and Caco-2 cells (Fig. 5C, D). Finally, HT-29 and Caco-2 CC cell-derived colonospheres lacking functional *ESR1* (derived from *siESR1-*transfected cells) showed intact ZO-1 expression compared to their *siCTRL*-transfected counterparts (Fig. 5E, E’, F, F′). Taken together, these observations indicate a role of ERα in promoting and supporting cell survival and metastasis in CC (Fig. 5G).

## Discussion

In this study, we identified the cellular mechanisms by which the nuclear receptor ERα drives metastasis and confers a worse prognosis of CRC patients (Fig. 1). The expression of ERα positively correlates with that of tumour promoters in CC. Activated ERα increases the level of active β-catenin (Figs. 2 and 3) and disrupts the tight junction proteins ZO-1 and Occludin (Figs. 4 and 5), leading to increased CC metastasis (Figs. 4 and 5).

Oestrogen receptors are well studied in breast and cervical cancers [2–4]. In colon cancer, ERβ plays an important role in limiting tumour progression and metastasis [5, 39, 40]. We have also previously reported that activating ERβ with the selective agonist ERB-041 reduced CC cell survival, induced apoptosis, and inhibited metastatic spread [27]. However, few studies have explored ERα expression levels in CC tissues. In our earlier report [24], we showed the prognostic relevance of ERα expression in CRC patients with higher ERα expression in tumour tissue than in normal mucosa. In addition, we established the importance of considering both ERα and ERβ protein expression for better predicting the prognosis of CRC patients [24].

Here, we investigated the correlation of ERα expression with that of tumour promoters in CRC. We noted a poorer prognosis in CRC patients with high ERα expression compared to the patients with low ERα expression (Fig. 1), which could be due to the significant correlations between ERα and both CysLT_1_R and β-catenin, all known tumour promoters [18, 20, 25, 28, 32, 35]. Our results were supported by mRNA expression data from public databases (Fig. 2C, D), which showed positive correlations between *ESR1* and both *CYSLTR1* and *CTNNB1* mRNA levels in CRC patients. Moreover, we have reported earlier that CysLT_1_R, and β-catenin expression are positively correlated in CRC patients and patients with high CysLT_1_R, and high nuclear β-catenin have poorer prognosis [22]. To strengthen our findings, we examined the expression of ERα in two different CC mouse models. *Cysltr1*^*−/−*^ mice, known to have a less aggressive phenotype, exhibited decreased ERα expression, while *Apc*^*Min/+*^ mice showed higher ERα expression (Fig. 2G, H). The *Apc*^*Min/+*^ mouse model exhibits activation of Wnt/β-catenin signalling, which promotes the translocation of β-catenin into the nucleus and positively correlates with CysLT_1_R expression as per our earlier report [32]. In addition, using both colon and breast cancer cells, *Kouzmenko* et al. have shown interaction between ERα and β-catenin via immunoprecipitation [41]. Taken together this indicate the possibility of an ERα/CysLT_1_R/Wnt signalling axis.

To maintain normal epithelial tissue integrity and cell polarity, tight junctions sustain cell-cell adhesion and regulates intercellular signalling [42]. Loss of tight junctions in a cluster of cancer cells in the primary tumour could result in dissemination and metastasis [42]. ZO-1 is a member of the tight-junction family of proteins (including Occludin) expressed in epithelial and endothelial cells and positively associated with β-catenin expression [43, 44]. Similar to ZO-1, loss of membrane β-catenin is associated with the migratory phenotype of cancer cells. Previously, the expression levels of the tight junction proteins ZO-1 and Occludin were reported to be reduced after oestrogen treatment, resulting in ERβ activation in human gut tissue [45]. *Zhou* et al. used both male and female gut tissues to highlight that ZO-1 is crucial for maintaining epithelial integrity in human gut tissue. Moreover, ZO-1 loss increases gut permeability and confers vulnerability to mucosal pathogens [45]. In a more recent study, *Li* et al. emphasized the metastasis-promoting role of ZO-1 in *KRAS*^*mut*^ CRC patients in a sex-specific manner [46].

We noted that pharmacological induction of ERα expression with a specific agonist, PPT, reduced the protein levels of ZO-1 and Occludin, which were restored using the specific antagonist AZD9496 (Fig. 4F, G). This is in coordination with the alteration noted in the β-catenin expression in the mRNA or protein level (Figs. 2I, J; 3C). Silencing of *ESR1* (by *siESR1* transfection) in CC cells also protected ZO-1 and Occludin expression. This was also reflected in the number of embryos with metastasis in the zebrafish model (Fig. 4C). Compared to injection of either AZD9496-treated cells or *siESR1*-transfected cells, injection of CC cells with reduced expression of tight junction proteins upon PPT treatment resulted in an increased number of embryos with tail vein metastasis (Figs. 4C and 5A). This indicates that tight junction disruption is a prerequisite step in ERα-mediated CC metastasis.

Taken together, our findings show that positive expression of ERα correlates with poor prognosis and metastasis in CRC patients. These findings highlight the potential therapeutic opportunities of blocking ERα expression in CRC [47]. However, further mechanistic studies are needed to analyse the pathway in more detail. The mechanistic insights provided here could be used as a framework for the development of precision therapeutic agents targeting ERα in metastatic CRC, particularly in cases where its expression is increased, such as cases in female patients with Lynch syndrome and breast or colorectal cancer.

### Supplementary Information

**Supplementary Material 1.**

**Supplementary Material 2.**
