# Correction: High Oestrogen receptor alpha expression correlates with adverse prognosis and promotes metastasis in colorectal cancer

**DOI:** 10.1186/s12964-024-01631-9

**Published:** 2024-05-02

**Authors:** Geriolda Topi, Shakti Ranjan Satapathy, Souvik Ghatak, Karin Hellman, Fredrik Ek, Roger Olsson, Roy Ehrnström, Marie-Louise Lydrup, Anita Sjölander

**Affiliations:** 1 Division of Cell and Experimental Pathology, Department of Translational Medicine, Lund University, Malmö, Sweden; 2 Chemical Biology & Therapeutics Group, Department of Experimental Medical Science, Lund University, Lund, Sweden; 3 Department of Pathology, Skåne University Hospital, Malmö, Sweden; 4 Division of Surgery, Skåne University Hospital, Malmö, Sweden; 5 Department of Endocrinology, Skåne University Hospital, Malmö, Sweden

**Correction: Cell Commun Signal 22, 198 (2024**)

10.1186/s12964-024-01582-1.

Following publication of the original article [1], the authors identified an error in the Fig. 5E. The representative image for ZO-1 expression in the HT-29 colonospheres (*siESR1* panel) was incorrect. The authors sincerely apologize for missing the mistake during the proofreading stage. The correct image is provided in this correction article. The correction does not affect the conclusions of the original research article.

The incorrect Fig. 5.

**Fig. 5:**
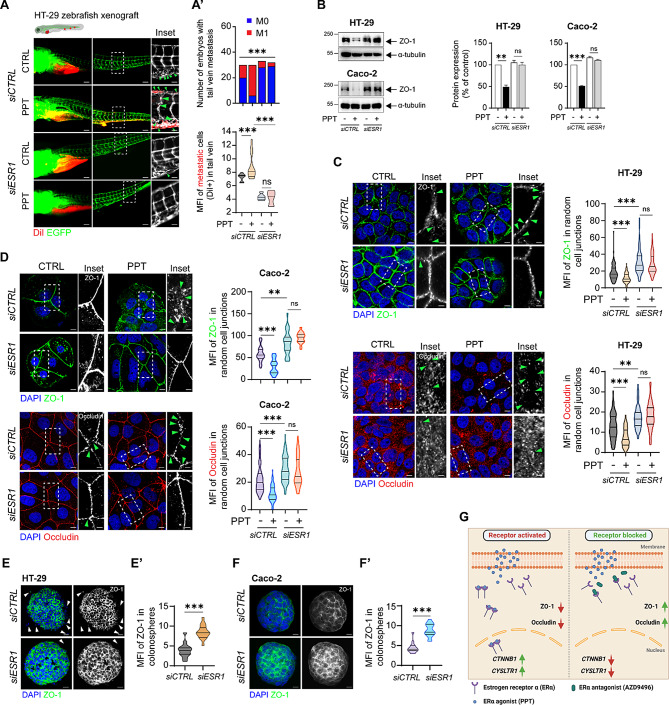
Functional absence of ERα inhibits colon cancer cell metastasis. DiI-labelled HT-29 cells transfected with either *siCTRL* or *siESR1* and treated with or without PPT for 48 h were injected into the perivitelline space of 2 dpf zebrafish embryos, and the embryos were incubated for 48 h. (**A**) Images showing the metastatic spread of HT-29 cells in the tail veins of zebrafish embryos in each group (*siCTRL*; CTRL, n = 30; PPT, n = 30; *siESR1*; CTRL, n = 33, PPT, n = 32). Graphs showing **A’**, the number of embryos with (M1, mets) or without metastasis (M0, no mets) in each group and **A’’**, the quantification of tail vein metastasis using the mean fluorescence intensity (MFI) of the embryos with metastasis (M1 group). Scale bars: full-size images; 10 μm, insets; 2 μm. The insets show the regions enclosed in the dotted lines in the full-size tail images. The arrows point to the metastatic foci and transendothelial migration of cancer cells. (**B**) Western blots showing the expression of the tight junction protein ZO-1 in HT-29 and Caco-2 cells transfected with either *siCTRL* or *siESR1* prior to PPT (40 nM) treatment. Graphs showing the densitometric analysis of alterations in protein expression as a percentage of the loading control (α-tubulin). The blots are representative of *n* = 3 independent experiments. (**C**) Immunofluorescence analysis of ZO-1 and Occludin expression in HT-29 cells transfected with either *siCTRL* or *siESR1* prior to treatment with the ERα agonist PPT (40 nM). Greyscale images (insets) showing representative regions of interest for ZO-1 and Occludin staining. Scale bars: full-size images; 5 μm, insets; 1 μm. Violin plots showing the mean fluorescence intensity of ZO-1 (*siCTRL* (CTRL, *n* = 105; PPT, *n* = 115), *siESR1* (CTRL, *n* = 108; PPT, *n* = 116)) and Occludin (*siCTRL* (CTRL, *n* = 105; PPT, *n* = 105), *siESR1* (CTRL, *n* = 107; PPT, *n* = 102)) in random cell-cell junctions. The arrows indicate gaps in ZO-1 or Occludin expression. (**D**) Immunofluorescence analysis of ZO-1 and Occludin expression in Caco-2 cells transfected with either *siCTRL* or *siESR1* prior to treatment with the ERα agonist PPT (40 nM). Greyscale images (insets) showing representative regions of interest for ZO-1 and Occludin staining. Scale bars: full-size images; 5 μm, insets; 1 μm. Violin plots showing the mean fluorescence intensity of ZO-1 and Occludin in random cell junctions. For ZO-1 staining in the *siCTRL*-transfected group (CTRL, *n* = 105; PPT, *n* = 105) and in the *siESR1*-transfected group (CTRL, *n* = 108; PPT, *n* = 105), random cell junctions were evaluated. For Occludin staining in the *siCTRL*-transfected group (CTRL, *n* = 105; PPT, *n* = 105) and in the *siESR1*-transfected group (CTRL, *n* = 102; PPT, *n* = 108), random cell junctions were evaluated. The arrows indicate gaps in ZO-1 or Occludin expression. Immunofluorescence analysis of ZO-1 in colonospheres derived from either *siCTRL* or *siESR1* transfected (**E**) HT-29 and (**F**) Caco-2 CC cells. Scale bars: 10 μm. Violin plots showing the mean fluorescence intensity of ZO-1 in random (**E’**) HT-29 (*siCTRL*, *n* = 30; *siESR1*, *n* = 32) or (**F’**) Caco-2 (*siCTRL*, *n* = 28; *siESR1*, *n* = 31) colonospheres. The MFIs of the indicated proteins were measured using ImageJ software (NIH, USA). (**G**) Graphical representation of the summary of the study. Upon binding to the agonist PPT, ERα dimerizes and shuttles into the nucleus. This upregulates the transcription of *CYSLTR1* and *CTNNB1*. In addition, it promotes metastasis by disrupting the tight junction proteins ZO-1 and Occludin. However, blocking the binding of PPT to ERα by employing an antagonist, AZD9496, prevents the activation and hence the dimerization of the receptor. This further leads to downregulation of *CYSLTR1* and *CTNNB1* and upregulation of the tight junction proteins ZO-1 and Occludin. The data are presented as the mean ± SEM of three experiments. P values were calculated with the chi-square test for A’ and unpaired Student’s *t* test for A’’, B-F

The correct Fig. 5.

**Fig. 5:**
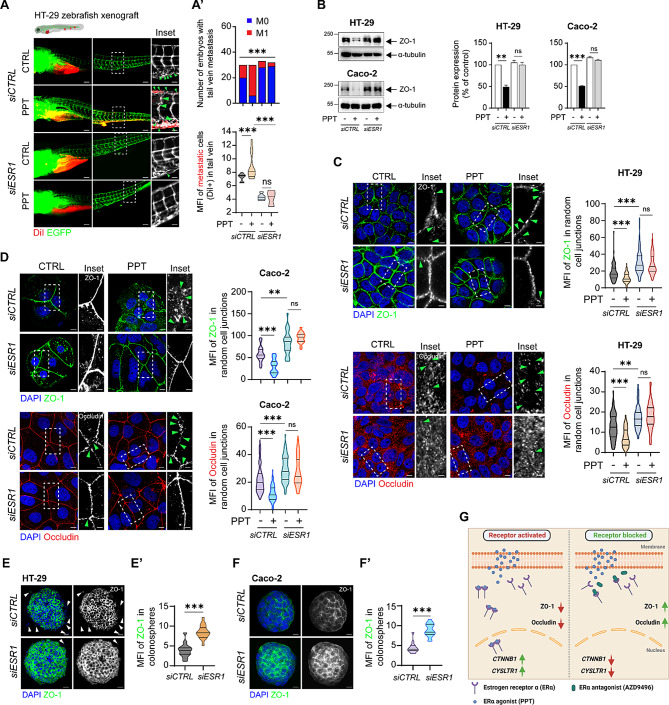
Functional absence of ERα inhibits colon cancer cell metastasis. DiI-labelled HT-29 cells transfected with either *siCTRL* or *siESR1* and treated with or without PPT for 48 h were injected into the perivitelline space of 2 dpf zebrafish embryos, and the embryos were incubated for 48 h. (**A**) Images showing the metastatic spread of HT-29 cells in the tail veins of zebrafish embryos in each group (*siCTRL*; CTRL, n = 30; PPT, n = 30; *siESR1*; CTRL, n = 33, PPT, n = 32). Graphs showing **A’**, the number of embryos with (M1, mets) or without metastasis (M0, no mets) in each group, and **A’’**, the quantification of tail vein metastasis using the mean fluorescence intensity (MFI) of the embryos with metastasis (M1 group). Scale bars: full-size images; 10 μm, insets; 2 μm. The insets show the regions enclosed in the dotted lines in the full-size tail images. The arrows point to the metastatic foci and transendothelial migration of cancer cells. (**B**) Western blots showing the expression of the tight junction protein ZO-1 in HT-29 and Caco-2 cells transfected with either *siCTRL* or *siESR1* prior to PPT (40 nM) treatment. Graphs showing the densitometric analysis of alterations in protein expression as a percentage of the loading control (α-tubulin). The blots are representative of *n* = 3 independent experiments. (**C**) Immunofluorescence analysis of ZO-1 and Occludin expression in HT-29 cells transfected with either *siCTRL* or *siESR1* prior to treatment with the ERα agonist PPT (40 nM). Greyscale images (insets) showing representative regions of interest for ZO-1 and Occludin staining. Scale bars: full-size images; 5 μm, insets; 1 μm. Violin plots showing the mean fluorescence intensity of ZO-1 (*siCTRL* (CTRL, *n* = 105; PPT, *n* = 115), *siESR1* (CTRL, *n* = 108; PPT, *n* = 116)) and Occludin (*siCTRL* (CTRL, *n* = 105; PPT, *n* = 105), *siESR1* (CTRL, *n* = 107; PPT, *n* = 102)) in random cell-cell junctions. The arrows indicate gaps in ZO-1 or Occludin expression. (**D**) Immunofluorescence analysis of ZO-1 and Occludin expression in Caco-2 cells transfected with either *siCTRL* or *siESR1* prior to treatment with the ERα agonist PPT (40 nM). Greyscale images (insets) showing representative regions of interest for ZO-1 and Occludin staining. Scale bars: full-size images; 5 μm, insets; 1 μm. Violin plots showing the mean fluorescence intensity of ZO-1 and Occludin in random cell junctions. For ZO-1 staining in the *siCTRL*-transfected group (CTRL, *n* = 105; PPT, *n* = 105) and in the *siESR1*-transfected group (CTRL, *n* = 108; PPT, *n* = 105), random cell junctions were evaluated. For Occludin staining in the *siCTRL*-transfected group (CTRL, *n* = 105; PPT, *n* = 105) and in the *siESR1*-transfected group (CTRL, *n* = 102; PPT, *n* = 108), random cell junctions were evaluated. The arrows indicate gaps in ZO-1 or Occludin expression. Immunofluorescence analysis of ZO-1 in colonospheres derived from either *siCTRL* or *siESR1* transfected (**E**) HT-29 and (**F**) Caco-2 CC cells. Scale bars: 10 μm. Violin plots showing the mean fluorescence intensity of ZO-1 in random (**E’**) HT-29 (*siCTRL*, *n* = 30; *siESR1*, *n* = 32) or (**F’**) Caco-2 (*siCTRL*, *n* = 28; *siESR1*, *n* = 31) colonospheres. The MFIs of the indicated proteins were measured using ImageJ software (NIH, USA). (**G**) Graphical representation of the summary of the study. Upon binding to the agonist PPT, ERα dimerizes and shuttles into the nucleus. This upregulates the transcription of *CYSLTR1* and *CTNNB1*. In addition, it promotes metastasis by disrupting the tight junction proteins ZO-1 and Occludin. However, blocking the binding of PPT to ERα by employing an antagonist, AZD9496, prevents the activation and hence the dimerization of the receptor. This further leads to downregulation of *CYSLTR1* and *CTNNB1* and upregulation of the tight junction proteins ZO-1 and Occludin. The data are presented as the mean ± SEM of three experiments. P values were calculated with the chi-square test for A’ and unpaired Student’s *t* test for A’’, B-F
