# Supplementary material for: High Oestrogen receptor alpha expression correlates with adverse prognosis and promotes metastasis in colorectal cancer
Source: Cell Commun Signal. 2024 Mar 28;22:198. doi: 10.1186/s12964-024-01582-1 (PMC10979551; doi:10.1186/s12964-024-01582-1)

Supplementary file

High Oestrogen Receptor Alpha Expression Correlates with Adverse Prognosis and Promotes Metastasis in Colorectal Cancer

Geriolda Topi[1,5#], Shakti Ranjan Satapathy[1#*], Souvik Ghatak[1], Karin Hellman[2], Fredrik Ek[2], Roger Olsson[2], Roy Ehrnström[3], Marie-Louise Lydrup[4], Anita Sjölander[1*]

[1]Cell pathology, Department of Translational Medicine, Lund University, Malmo, Sweden.

[2]Chemical Biology & Therapeutics Group, Department of Experimental Medical Science, Lund University, Lund, Sweden

[3] Department of Pathology, Skåne University Hospital, Malmö, Sweden

[4]Division of Surgery, Skåne University Hospital, Malmö, Sweden

[5]Department of Endocrinology, Skåne University Hospital, Malmö, Sweden

#Equal contributions

*Correspondence: Dr. Shakti Ranjan Satapathy, Division of Cell and Experimental Pathology, Department of Translational Medicine, Lund University, Malmö, Sweden. E-mail: shakti_ranjan.satapathy@med.lu.se

Professor Anita Sjölander, Division of Cell and Experimental Pathology, Department of Translational Medicine, Lund University, Skåne University Hospital, Malmö, Sweden. E-mail: Anita.Sjolander@med.lu.se

Method:

Wound healing assay

To test the migration capacity of HT-29 CC cells *in vitro*, the wound-healing assay was performed as mentioned earlier [1, 2]. Ibidi wound-healing inserts were used as per the manufacturer's instructions (80209, IBIDI, GMBH, Germany). HT-29 CC cells were treated with PPT (40 nM) with or without AZD9496 pre-treatment (0.3 nM, 30 min). Similarly, *siESR1* or *siCTRL* transfected HT-29 colon cancer cells with or without PPT (40 nM) treatment were used for the assay. The percentage of wound closure was measured using ImageJ software (NIH, USA).

References:

[1] S.R. Satapathy, A. Sjolander, Cysteinyl leukotriene receptor 1 promotes 5-fluorouracil resistance and resistance-derived stemness in colon cancer cells, Cancer Lett 488 (2020) 50-62.

[2] G. Topi, S.R. Satapathy, P. Dash, S. Fred Mehrabi, R. Ehrnstrom, R. Olsson, M.L. Lydrup, A. Sjolander, Tumour-suppressive effect of oestrogen receptor beta in colorectal cancer patients, colon cancer cells, and a zebrafish model, J Pathol 251(3) (2020) 297-309.

Figure legends:

Supplementary fig. S1 Flow chart.

Flow chart showing the stratification of colon cancer patients used in the study involving two cohorts, the Female cohort (n = 333), and the Malmo cohort 1990 (n = 120).

Supplementary fig. S2 ERα stimulation promotes wound healing in colon cancer cells.

A, Images showing wound closure in HT-29 colon cancer (CC) cells compared between 0 h and 24 h. Cells were treated with PPT (ERα specific agonist, 40 nM) with or without AZD9496 pre-treatment (ERα specific antagonist, 0.3 nM for 30 min before the PPT treatment) for 24 h and images were captured at 0 h and 24 h. The percentage of wound closure is represented in the bar graph.

B, HT-29 CC cells were transfected with *siCTRL* or *siESR1* followed by wound healing assay with or without PPT treatment (ERα specific agonist, 40 nM). Images were captured at 0 h and 24 h. The percentage of wound closure is represented in the bar graph.

The data are presented as the mean ± SEM (n = 3 independent experiments). P values < 0.5 were considered significant and were calculated using an unpaired Student's *t* test.

Supplementary fig. S1

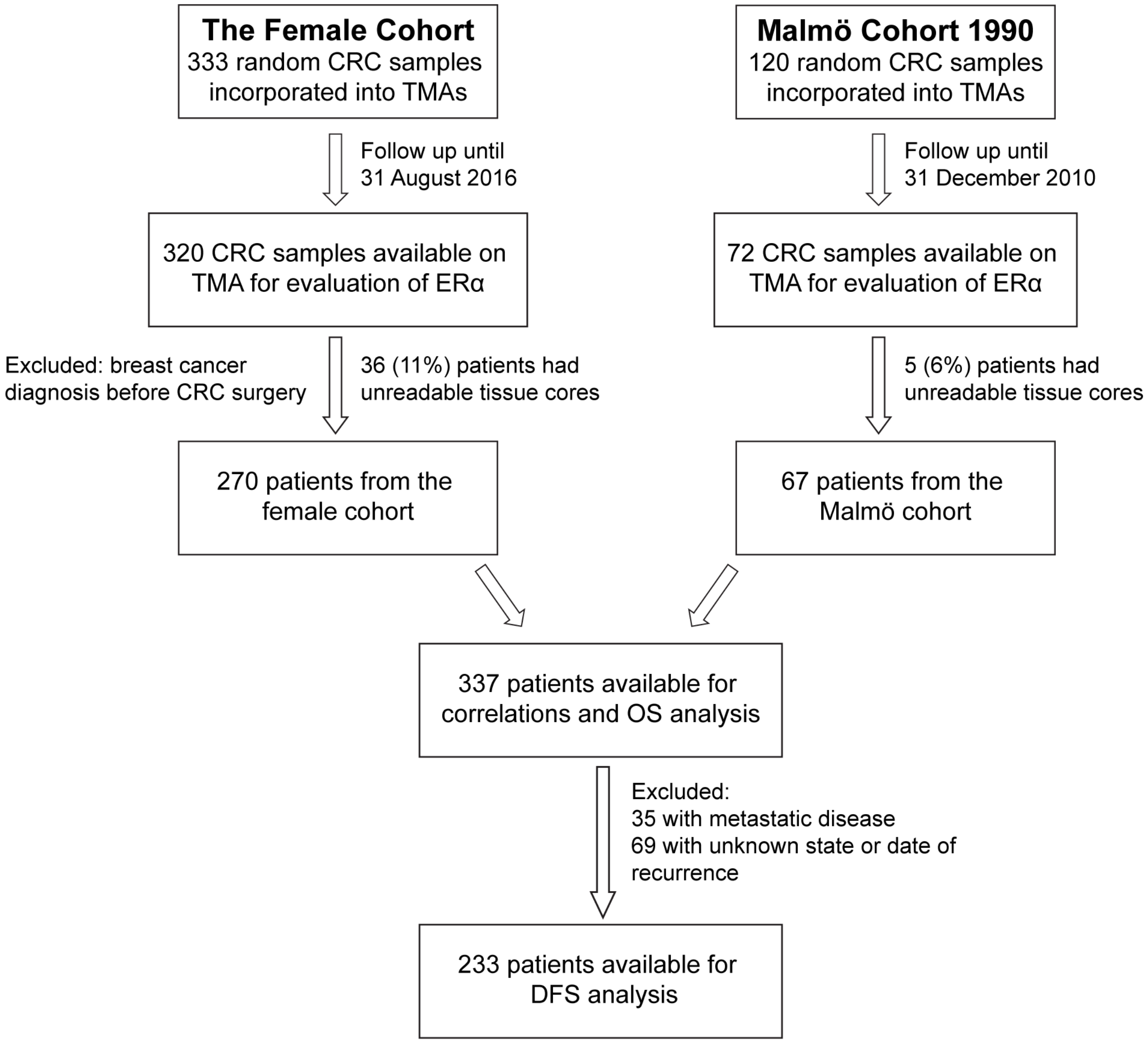

Supplementary fig. S2

A

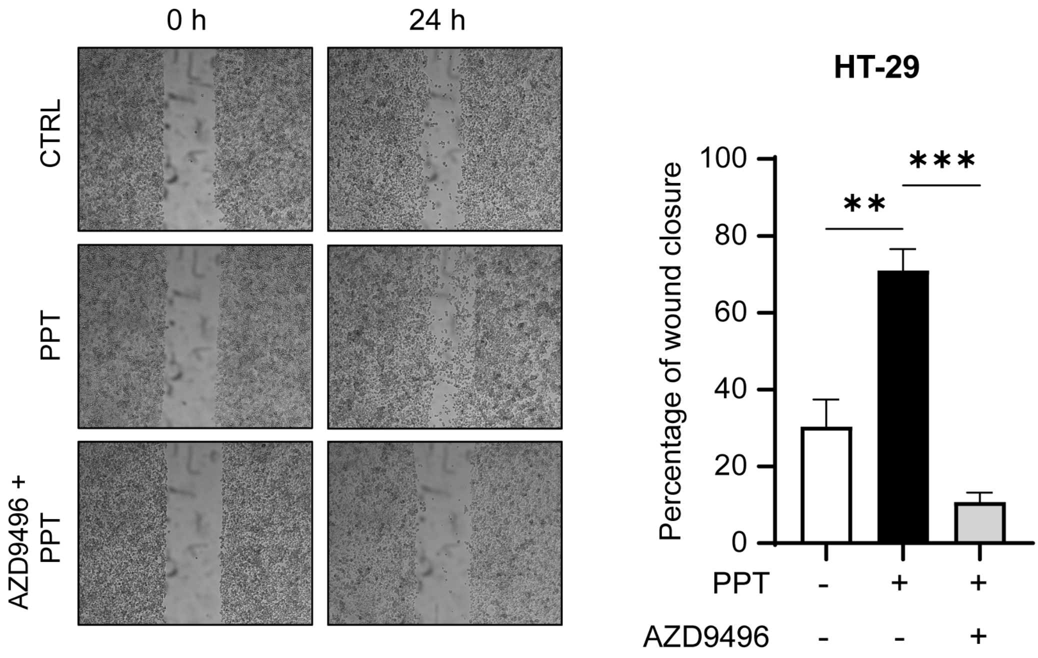

B

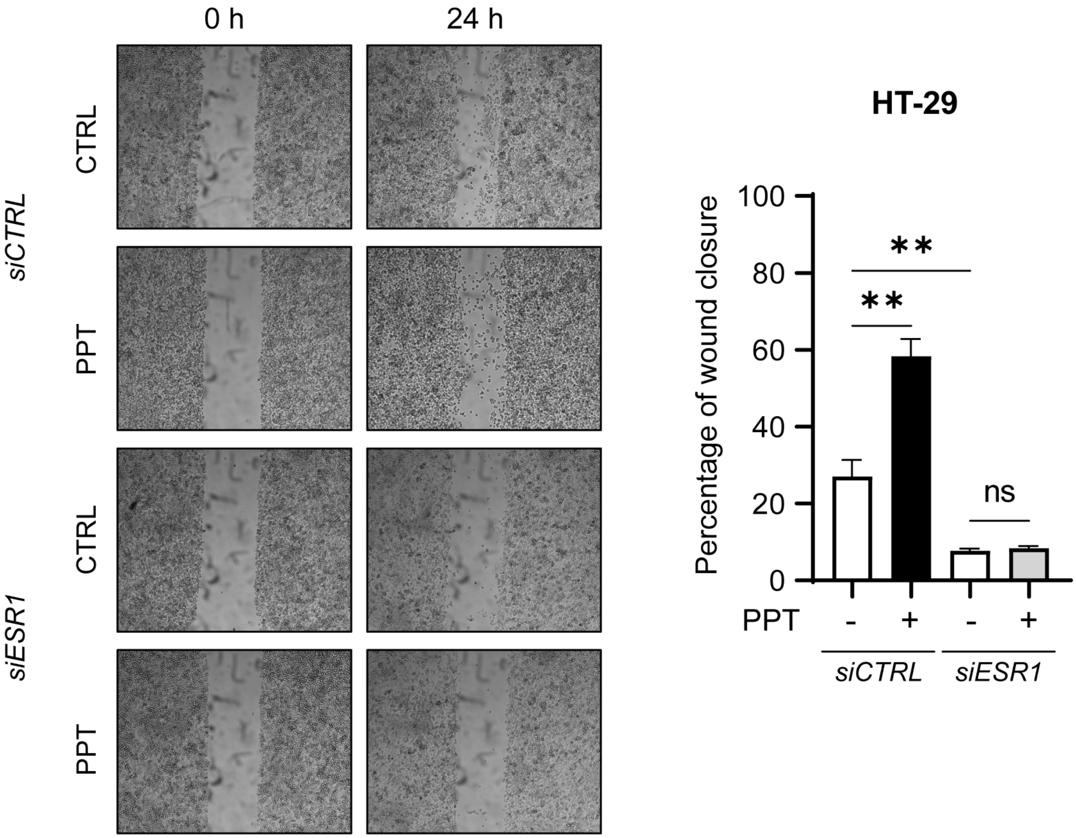

Supplement: Supplementary file 1 — Supplementary Material 1. [file 12964_2024_1582_MOESM1_ESM.pdf]
