# Supplementary material for: High Oestrogen receptor alpha expression correlates with adverse prognosis and promotes metastasis in colorectal cancer
Source: Cell Commun Signal. 2024 Mar 28;22:198. doi: 10.1186/s12964-024-01582-1 (PMC10979551; doi:10.1186/s12964-024-01582-1)

Supplementary file 2

Full unedited blots

Figure 1A

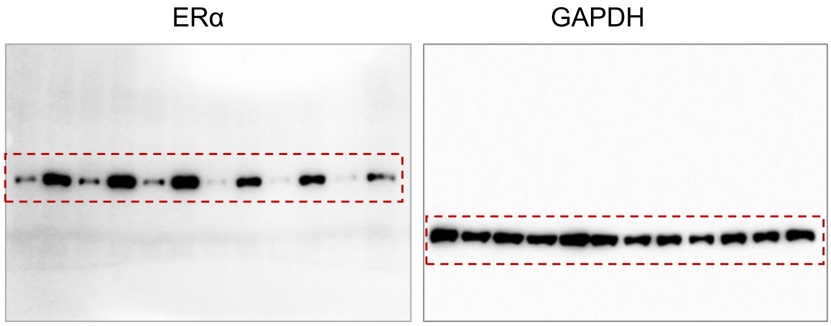

Figure 3B

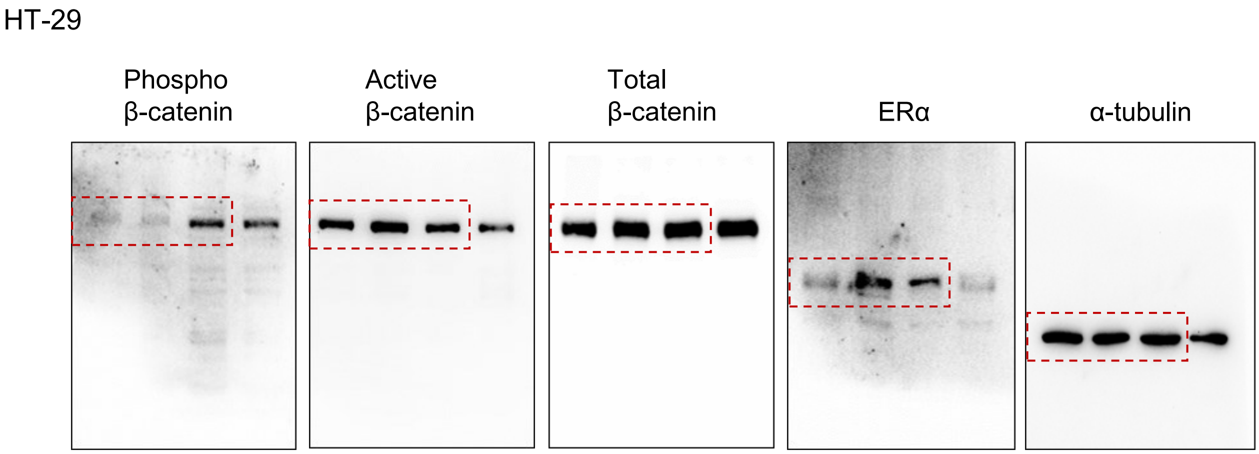
HT-29
Phospho β-catenin
Active β-catenin
Total β-catenin
ERα
α-tubulin

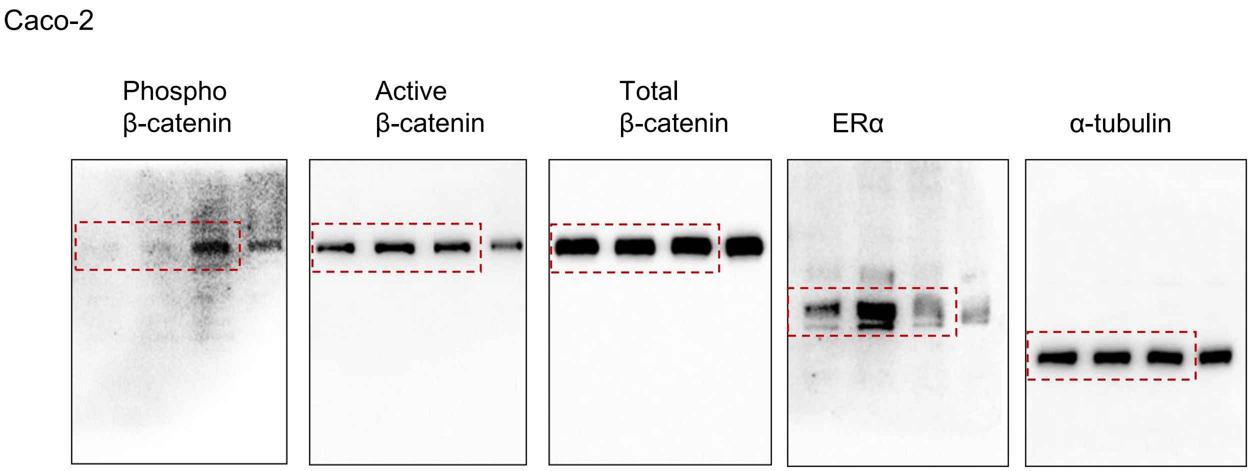
Caco-2
Phospho β-catenin
Active β-catenin
Total β-catenin
ERα
α-tubulin

Figure 3D

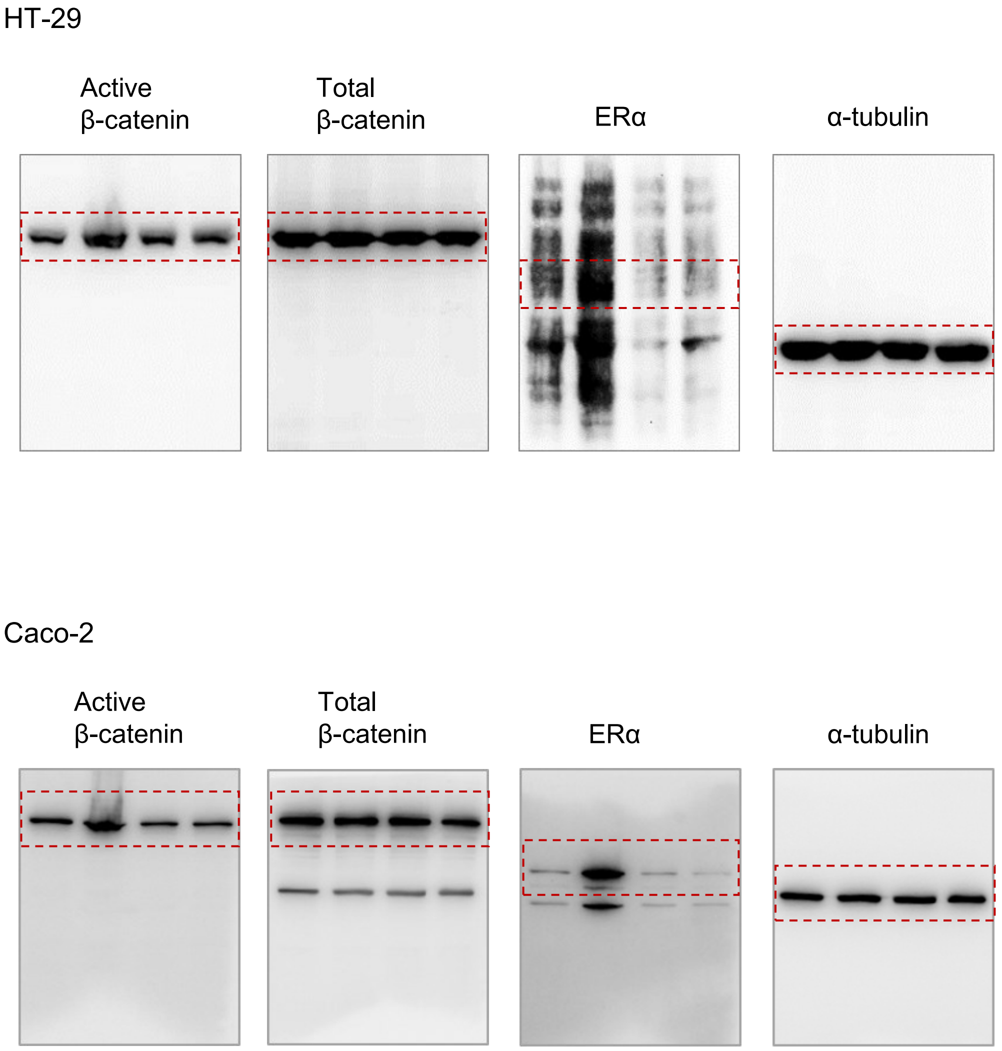
HT-29
Active β-catenin
Total β-catenin
ERα
α-tubulin
Caco-2
Active β-catenin
Total β-catenin
ERα
α-tubulin

Figure 4E

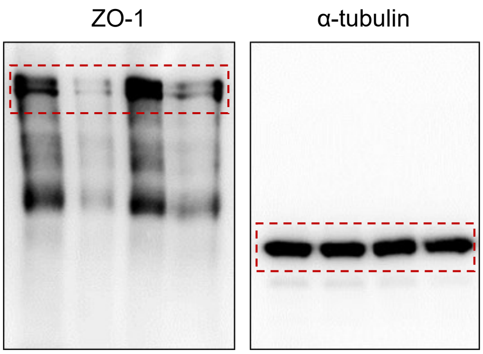

Figure 5B

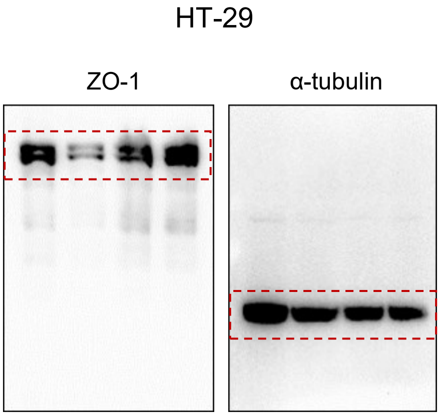

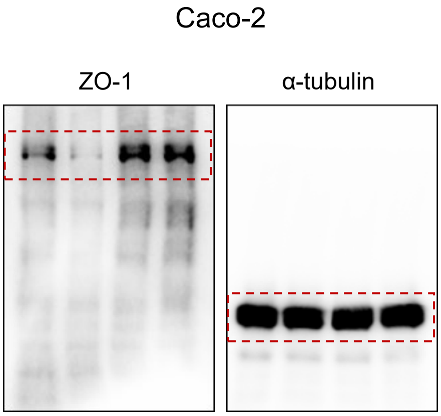

Supplement: Supplementary file 2 — Supplementary Material 2. [file 12964_2024_1582_MOESM2_ESM.pdf]
